# Gender Differences in 30-Day Re-Hospitalization Among Medicare Beneficiaries With Alzheimer’s and Dementia

**DOI:** 10.1093/geroni/igaa057.2444

**Published:** 2020-12-16

**Authors:** Andrea Gilmore-Bykovskyi

**Affiliations:** University of Wisconsin-Madison, Madison, Wisconsin, United States

## Abstract

Hospitalization is associated with accelerated cognitive decline for persons with Alzheimer’s disease and related dementia (ADRD), which disproportionately impacts women. Persons with ADRD are also at higher risk for 30-day rehospitalization, which may compound the impact of hospitalization-related exposures that precipitate decline. Evidence surrounding the intersections between gender and rehospitalization risk among diverse, representative populations with ADRD are lacking. This retrospective cohort study used a 100% national sample of Medicare beneficiaries with a diagnosis of ADRD and qualifying index hospitalization in 2014 (n= 1,033,144 unique beneficiaries and 1,672,238 unique stays). The primary outcome was rate of 30-day rehospitalization by gender and race. Within each racial group, men have higher rehospitalization rates than women: 2.6% higher among white men, 1.7% among African American men, and 2.6% higher among other racial/ethnic minorities. Findings highlight the importance of elucidating mechanisms underlying gender differences in hospital utilization and subsequent impact on cognitive decline.

